# Factors Influencing Resistivity Inversion for CO_2_ Geological Storage Zones: A Quantitative Study

**DOI:** 10.3390/s25061796

**Published:** 2025-03-14

**Authors:** Chenguang Wang, Tianyang Li, Tao Yu, Xiao Feng, Hanghang Liu, Bingrui Du, Yichun Yang, Linjun Yang, Yaxuan Luo

**Affiliations:** 1 School of Resources and Safety Engineering, Chongqing University, Chongqing 400044, China; 202220021013@stu.cqu.edu.cn (C.W.); 202320021009@stu.cqu.edu.cn (T.Y.); 20223497@stu.cqu.edu.cn (Y.Y.); 20223886@stu.cqu.edu.cn (L.Y.); 20222694@stu.cqu.edu.cn (Y.L.); 2 State Key Laboratory of Coal Mine Disaster Dynamics and Control, Chongqing University, Chongqing 400044, China; 3 School of Electrical Engineering, Chongqing University, Chongqing 400044, China; loydf123@foxmail.com (X.F.); 202111131215@cqu.edu.cn (H.L.); 4 Institute of Geophysical and Geochemical Exploration, Chinese Academy of Geological Sciences, Langfang 065000, China

**Keywords:** cross-borehole electrical resistivity, resistivity inversion, CO_2_ storage monitoring, simulation

## Abstract

This study establishes a homogeneous half-space and a horizontally layered two-layer background stratigraphy model using cross-borehole electrical resistivity tomography (ERT) based on an incomplete Gauss–Newton (IGN) method to investigate the resistivity inversion characteristics of CO_2_ storage zones. The effects of storage zone volume ($V_{{\mathrm{CO}}_{2}}$), storage zone resistivity ($\rho_{{\mathrm{CO}}_{2}}$), background formation resistivity ($\rho_{f}$), and CO_2_ diffusion on inversion results were systematically analyzed, and the mechanisms underlying the influence of different parameters on inversion imaging were explored. The results indicate that an increase in the $V_{{\mathrm{CO}}_{2}}$ significantly affects the inverted resistivity. The $\rho_{{\mathrm{CO}}_{2}}$ can be well inverted within a certain range, but inversion accuracy decreases once the resistivity exceeds a threshold. The $\rho_{f}$ is a critical factor influencing inversion results; as the $\rho_{f}$ increases, the inverted resistivity values rise markedly, although this effect exhibits an upper limit. The study also uncovers the exponential nature of CO_2_ diffusion in the storage zone, where diffusion leads to exponential changes in resistivity and the delineation of the diffusion zone is enhanced by comparing pre- and post-injection resistivity differences. These findings offer valuable insights for CO_2_ storage monitoring, contributing to both safety assessments and the evaluation of storage stability in geological sequestration.

## 1. Introduction

As global efforts to reduce greenhouse gas emissions intensify, carbon capture and storage (CCS) has emerged as a crucial technology for addressing the growing challenge of atmospheric CO_2_ levels. The geological storage of CO_2_ provides a sustainable solution to storing large quantities of COCO_2_ underground, preventing its release into the atmosphere and thereby reducing the greenhouse effect. Moreover, geological storage is a long-term solution that can sequester CO_2_ for centuries, helping to stabilize the global climate and meet international carbon reduction targets [1]. However, a significant challenge remains: ensuring the long-term stability and safety of CO_2_ storage in these reservoirs over extended periods. This challenge necessitates the development and implementation of effective monitoring strategies that can continuously assess the storage zones, ensuring the integrity of the sequestration process and minimizing potential environmental risks [2,3,4].

Continuous monitoring of changes in the subsurface distribution of CO_2_ is crucial for evaluating the effectiveness and safety of the sequestration process. Reservoir geophysical imaging provides insights into the properties of the formations, with resistivity serving as one of the most critical parameters. The resistivity of geological formations is influenced by the conductivity of rocks and minerals, which varies across the Earth’s crust. These differences in conductivity make resistivity an important indicator for detecting changes in subsurface material structures [5]. Electrical resistivity tomography (ERT) is a widely adopted geophysical technique that offers high sensitivity to variations in the resistivity of pore fluids and the different types of rocks present in the subsurface [6,7,8]. Several advantages of ERT include its ease of implementation, non-invasive nature, cost-effectiveness, high spatial resolution, and relatively high efficiency [9,10,11,12,13]. The injection of high-resistivity CO_2_ could induce significant changes in the resistivity of the formation. This sensitivity to resistivity contrasts between rocks, pore fluids, and injected CO_2_ make ERT a powerful tool for tracking CO_2_ movement and migration within the reservoir. By measuring and analyzing changes in physical parameters such as resistivity before and after injection, it becomes possible to effectively estimate variations in CO_2_ within the storage zone, which also provides valuable information on the migration pathways and spatial distribution of CO_2_ in the reservoir over time.

Electrical resistivity tomography (ERT) offers detailed imaging of subsurface resistivity distributions, leveraging its high sensitivity to changes in liquid and gas saturation to monitor geological CO_2_ storage effectively. Lochbühler et al. [14] introduced a probabilistic inversion method that delivers robust CO_2_ distribution estimates by providing the full posterior probability density function of the saturation field, enriched by rock physics parameters linking resistivity to saturation. Breen et al. [15] demonstrated ERT’s ability to accurately map the shape, size, and position of CO_2_ plumes in a brine-saturated porous medium, though it overestimated brine saturation, highlighting data quality challenges over time. Nakatsuka et al. [16] confirmed that CO_2_ injection into brine-saturated zones increases resistivity, with clay presence reducing conductivity, based on lab and well-logging data from the Nagaoka site. Jia et al. [17] and Al et al. [18] enhanced inter-well ERT systems through optimized electrode arrays, successfully locating CO_2_-bearing zones, though discrepancies between ERT-imaged and -modeled sequestration zone shapes suggest a need for improved modeling and calibration.

Recent advances in multimodal monitoring underscore the potential of electrical resistivity tomography (ERT) when integrated with advanced sensor systems and complementary geophysical methods. At the Ketzin CO_2_ pilot site, integrated seismic–ERT monitoring utilized high-stability electrode arrays—designed with corrosion-resistant materials and optimized contact impedance—to achieve a 20% improvement in plume boundary definition accuracy compared to single-method approaches [19]. The CaMI_FRS trials showcased fiber-optic integrated ERT sensors, leveraging distributed sensing capabilities to enable joint inversion with cross-well electromagnetic methods, resulting in a 15% enhancement in CO_2_ saturation mapping resolution [20]. Further innovations in sensor design for extreme environments include cryogenic-grade electrodes, engineered with low-temperature conductive alloys for permafrost monitoring at −40 °C [21], and refractory sensor packages, featuring thermally resilient coatings to maintain measurement fidelity in 1200 °C molten oxide conditions [22]. These sensor-driven advancements not only improve ERT’s sensitivity and spatial resolution but also expand its applicability as a standalone and hybrid monitoring solution for carbon sequestration, aligning with the evolving demands of subsurface sensing technologies.

At present, significant progress has been made in the application of ERT technology in CO_2_ storage projects [23,24,25]. For example, in the Ketzin pilot CO_2_ sequestration project in Germany, both borehole-to-surface and inter-well ERT measurements confirmed that CO_2_ injection generates significant electrical signals at subsurface electrodes. This finding facilitates the real-time monitoring of the electrode potential, enhancing the capability to track changes in the subsurface environment during CO_2_ sequestration [24,26]. In addition, the joint inversion of seismic full-waveform inversion (FWI) and inter-well ERT provided three-dimensional models of geophysical parameters and their temporal variations. The real data from the Ketzin pilot project were used to test this joint inversion approach, revealing that CO_2_ injection resulted in a twofold increase in the resistivity of the sequestration zone [27,28]. Similarly, in the CaMI project in Canada, permanently installed monitoring electrodes successfully detected small amounts of injected CO_2_. The CO_2_ saturation derived from inversion showed a strong correlation with the actual amount of CO_2_ injected at the field research station, highlighting the reliability of the monitoring system for detecting and quantifying CO_2_ sequestration [29,30,31]. At the Svelvik CO_2_ pilot site, four monitoring wells were drilled to a depth of 100 m, each equipped with 16 electrodes. The impacts of CO_2_ injection on formation saturation and pore pressure were effectively monitored, further demonstrating the capability of ERT for the real-time subsurface monitoring of CO_2_ storage [32].

Despite these advancements, current research still lacks quantitative evaluations of the imaging performance of the resistivity method in CO_2_ storage zones. To address this gap, our study isolates the intrinsic coupling of CO_2_ storage parameters ($V_{{\mathrm{CO}}_{2}}$, $\rho_{{\mathrm{CO}}_{2}}$, and $\rho_{f}$) with inversion signatures using a fixed-geometry approach. While operational factors like electrode spacing and depth sensitivity have been systematically addressed in prior work [33,34], this analysis decouples geological influences from acquisition configurations to highlight core petrophysical trends. This study uses a resistivity inversion simulation method based on an IGN algorithm to analyze the effects of various factors such as storage zone volume, resistivity, background resistivity, and CO_2_ diffusion on resistivity inversion results. We investigate the influence of these factors on inversion outcomes and identify the extent of CO_2_ diffusion regions. The findings from this study are expected to provide valuable insights and practical guidance for the monitoring of CO_2_ sequestration in future geological storage projects, thereby improving the efficiency and accuracy of CO_2_ monitoring systems.

## 2. Methods

The resistivity inversion is a widely used computational technique in geophysical exploration, which enables the estimation of subsurface resistivity distributions. This technique involves measuring potential differences between surface or subsurface electrodes and applying known supply currents to infer the resistivity properties of underground media. The process typically aims to minimize the discrepancy between observed data and theoretical models, leveraging mathematical models and algorithms to reconstruct the subsurface resistivity distribution [35].

The inversion methods can be broadly categorized into two types: linear and nonlinear [36]. The key aspect is the establishment of a mathematical relationship between the observed data and the geological model parameters. The linear inversion method, using parameter substitution and Taylor series expansion, transforms nonlinear inverse problems into linear ones. These methods are particularly advantageous due to their excellent convergence behaviors, especially in multidimensional problems, and have thus become one of the most robust, widely applied, and effective approaches in geophysical practice. However, linear inversion methods often make simplifying assumptions that may not fully capture the complexities of real-world geological systems. In contrast, nonlinear inversion methods offer distinct advantages, as they are better suited to handle the inherent complexities and nonlinearity of most geophysical problems. These methods avoid the common issue of converging to local minima during iterative inversion, thereby providing more reliable solutions for complex geological scenarios, which is essential for accurately modeling subsurface structures, particularly in cases where the relationships between the observed data and geological properties are highly nonlinear.

In this study, we propose a resistivity inversion method based on the IGN method to investigate the inversion properties of the CO_2_ geological storage area. The main innovation of this method is its efficiency and stability in dealing with large-scale inversion problems, which significantly reduces the amount of calculations compared with traditional methods. In addition, we systematically analyzed the effects of CO_2_ storage area volume ($V_{{\mathrm{CO}}_{2}}$), storage area resistivity ($\rho_{{\mathrm{CO}}_{2}}$), background formation resistivity ($\rho_{f}$), and CO_2_ diffusion on the inversion results. The influence mechanism of these parameters on inversion imaging is revealed. These findings provide new theoretical support and technical guidance for the monitoring of CO_2_ geological sequestration.

Therefore, this study adopts a nonlinear inversion approach based on the finite element [37] forward modeling of resistivity imaging. A target function is constructed using the IGN method, with detailed derivations of the data term and model term within the target function. Additionally, a resistivity inversion workflow is established, providing a robust theoretical foundation for tackling resistivity inversion problems and enhancing the reliability and accuracy of subsurface resistivity estimations.

### 2.1. Solution of the Objective Function

The IGN method [38] is an improved version of the standard Gauss–Newton algorithm designed to address some of the computational challenges associated with large-scale inverse problems. In the standard Gauss–Newton method, each iteration involves solving a linear system where the Hessian matrix is approximated by the Jacobian matrix. While this approach is effective, it can be computationally expensive and prone to instability, particularly when dealing with large-scale matrices. The IGN method improves upon this by incorporating a relaxation strategy wherein the solution to the linear system is approximated rather than solved exactly during each iteration. This approximation significantly reduces computational costs while maintaining convergence, making the method particularly suitable for large-scale inverse problems where efficiency is a priority.

#### 2.1.1. Construction of the Objective Function

Resistivity inversion is a process aimed at determining the subsurface electrical structure of a target body based on known measured potential data. The core idea behind this process is as follows: through an iterative approach, the electrical parameter configuration of the model is gradually adjusted. Forward modeling algorithms are applied to generate corresponding predictive data, and the simulated response of these predictions is then compared with the observed data. If the predicted response closely matches the observed data, it can be inferred that the geoelectrical model based on the predicted data is a good approximation of the actual subsurface structure.

However, in resistivity inversion, the number of available observation data points is much smaller than the scale of the model parameters. This characteristic results in an underdetermined inversion problem, where multiple solutions may satisfy the observed data, leading to ambiguity in the inversion results. Moreover, the presence of data errors and noise can make the inversion ill posed. To address these challenges, it is necessary to apply additional constraints to the inversion process, which can help to regularize the inversion, transforming it into a well-posed optimization problem. By formulating resistivity inversion as a regularized optimization problem, the inversion process becomes more stable and reliable, enabling a more accurate reconstruction of subsurface resistivity distributions. In this study, the objective function can be expressed as follows:

(1) $$\phi \left(m\right)=\phi_{d}+\lambda \phi_{m}$$

where *m* represents the model parameters, $\phi_{d}$ refers to the data terms, $\phi_{m}$ corresponds to the model terms, and λ is the regularization parameter.

#### 2.1.2. Data and Model Terms

Based on previous studies and incorporating the finite element discretization method used in the forward solving process, the point-source potential distribution equation can be formulated as follows [39]:

(2) $$▽\cdot \left(\sigma ▽\varphi \right)=-I\delta \left(x-x_{0}\right)\delta \left(y-y_{0}\right)\delta \left(z-z_{0}\right)$$

It can be rewritten as the variational form of the following matrix equation:

(3) A(σ)φ=q

where φ represents the potential distribution at the tetrahedral grid nodes, while σ denotes the conductivity distribution vector within the region. *A* is defined as the forward operator in the resistivity space. Based on the theory of resistivity forward modeling, it can be observed that the matrix *A* is a large sparse matrix that is closely related to the grid discretization and the internal conductivity distribution of the model. *q* is the right-hand side term containing the point-source distribution.

The observation data vector *d* is a subset of the potential distribution vector φ at all grid nodes within the study area:

(4) $$d=Q\varphi =QA^{-1}\left(\sigma \right)q$$

where *Q* is the mapping matrix from φ to *d*. The objective function of least-squares inversion data is obtained using the Hilbert space approximation:

(5) $$\phi_{d}={∥C_{d}\left(d\left(m\right)-f\left(m\right)\right)∥}^{2}$$

where d(m) is the predicted data vector, which is obtained through forward modeling, f(m) is the observed data vector, and $C_{d}$ is the data weighting matrix. $C_{d}$ is selected as a diagonal matrix, i.e., $C_{d}=diag\left(\frac{1}{\varepsilon_{i}}\right)$, where $\varepsilon_{i}$ represents the error of the *i*-th observed data point.

The data term fit is expressed as the root mean square error (RMS) and is given by the following:

(6) $$RMS=\sqrt{\frac{1}{N}\sum {\left(\frac{d_{i}-f_{i}\left(m\right)}{\varepsilon_{i}}\right)}^{2}}$$

where *N* is the number of measurements, and the objective variance should be 1 (following a chi-square distribution).

In the calculation of the model term, to construct a smoother model structure, the difference between the model parameters and their adjacent parameters is minimized. The specific mathematical expression is as follows:

(7) $$\phi_{m}={∥C(m-m_{0})∥}^{2}$$

where $m_{0}$ is the initial model value, and *C* is the model weighting matrix.

The Gauss–Newton method is used to transform Equation (1) into the normal equation form:

(8) $$\left(J^{T}C_{d}^{T}C_{d}J+\lambda C^{T}C\right)△m=-J^{T}C_{d}^{T}C_{d}\left(d-f\left(m\right)\right)-\lambda C^{T}C\left(m-m_{0}\right)$$

Let

(9) $$H=J^{T}C_{d}^{T}C_{d}J+\lambda C^{T}C$$

(10) $$g=J^{T}C_{d}^{T}C_{d}\left(d-f\left(m\right)\right)-\lambda C^{T}C\left(m-m_{0}\right)$$

Thus, Equation (10) can be simplified as

(11) H△m=−g

where *H* is the Hessian matrix and *g* is the gradient of the objective function. During the inversion process, the Jacobian matrix $J=\frac{\partial d(m)}{\partial m}$ needs to be computed and stored. However, due to the large amount of data involved in resistivity inversion imaging, the size of the Jacobian matrix *J* becomes very large. As a result, directly computing and storing the matrix is avoided.

Haber [40] represents the Jacobian matrix *J* in the following form:

(12) $$J=QA^{-1}B$$

where $B=\frac{\partial (A(m)\varphi )}{\partial m}$ is the matrix closely related to the grid discretization of the study model and the internal conductivity distribution, since both *Q* and *B* are sparse matrices that can be stored in a compressed format [41] to reduce the data size of the Jacobian matrix.

The approximate solution to Equation (11) is obtained under the condition of effectively reducing computation time without affecting the resistivity inversion results.

### 2.2. ERT Inversion Process

**Figure 1:**
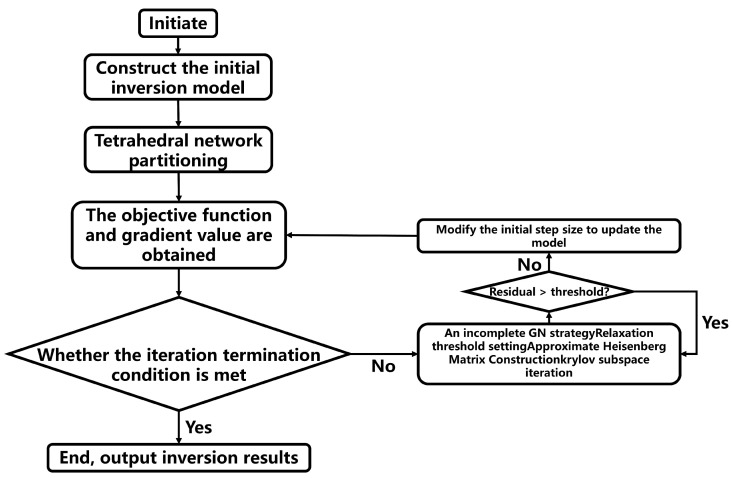
Iterative resistivity inversion process based on FEFM.

The resistivity inversion process based on finite element forward modeling (FEFM) is an iterative optimization procedure, where the forward and inversion problems are interwoven and solved iteratively (Figure 1). The process begins by defining the initial model, performing a tetrahedral discretization of the study area, and setting the iteration stopping criteria. Once the initial conditions are established, the following steps are carried out:

1. The model is discretized using tetrahedral elements, and an initial guess for the conductivity distribution is set. The roughness matrix of the model is computed to understand the initial model’s variations.
2. A forward modeling step is performed to simulate the potential distribution based on the current model. This step predicts the data, which are compared to the observed data.
3. Objective Function and Gradient Calculation: The objective function, representing the difference between the observed and predicted data, is calculated. The gradient of this function is also computed to determine the direction in which the model needs to be updated.
4. The inversion process is iteratively refined. If the value of the objective function falls below a predefined threshold or if the current iteration count exceeds the maximum number of allowed iterations, the process is terminated. Otherwise, the following steps are undertaken.
5. The initial step size is adjusted, and the sensitivity matrix is recalculated. The model parameters are updated based on the inversion iteration formula.
6. The updated model undergoes another forward response calculation, and the inversion process continues with the next round of iterations. Through this iterative process, the model parameters are refined, progressively improving the fit between the predicted and observed data until the stopping criteria are met.

## 3. Inversion Model Construction

**Figure 2:**
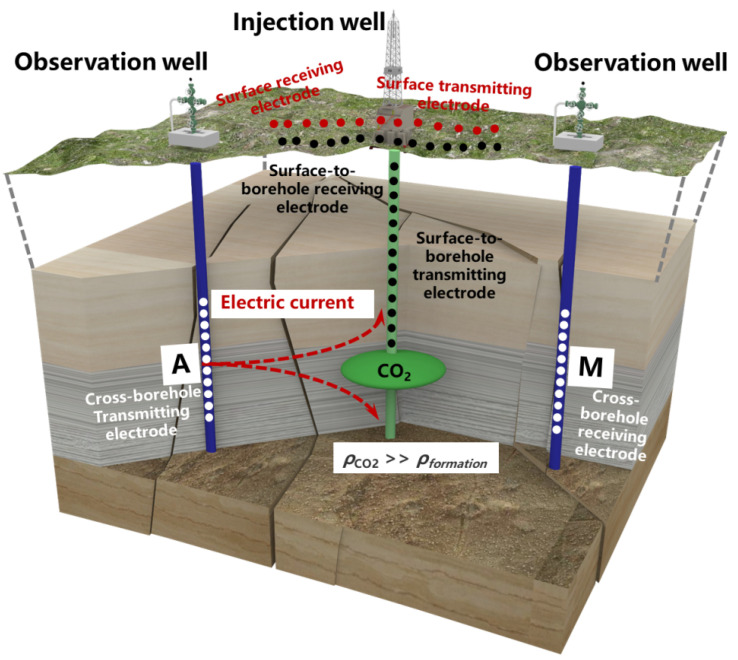
Schematic diagram of surface, surface-to-borehole, and cross-borehole ERT measurements.

Previous studies have shown that surface ERT imaging is effective for shallow resistivity imaging, where current and potential electrodes are placed on the surface. However, when the measurement depth is too large, the current must travel along a longer path to reach the target area [42,43]. This results in a rapid decay of the electric field strength and a significant reduction in resolution. Existing research studies suggest that certain ERT imaging techniques, such as the cross-borehole measurement shown in Figure 2 [39], can effectively shorten the current path and enhance the concentration of the electric field, thereby improving signal strength and resolution. Additionally, this method allows for the direct observation of resistivity changes across the boreholes, particularly when the target area, such as a CO_2_ storage zone, exhibits high-resistivity characteristics (Figure 2). This allows for a more sensitive detection of disturbances and changes in the electric field, making it a practical tool for monitoring CO_2_ storage sites.

**Figure 3:**
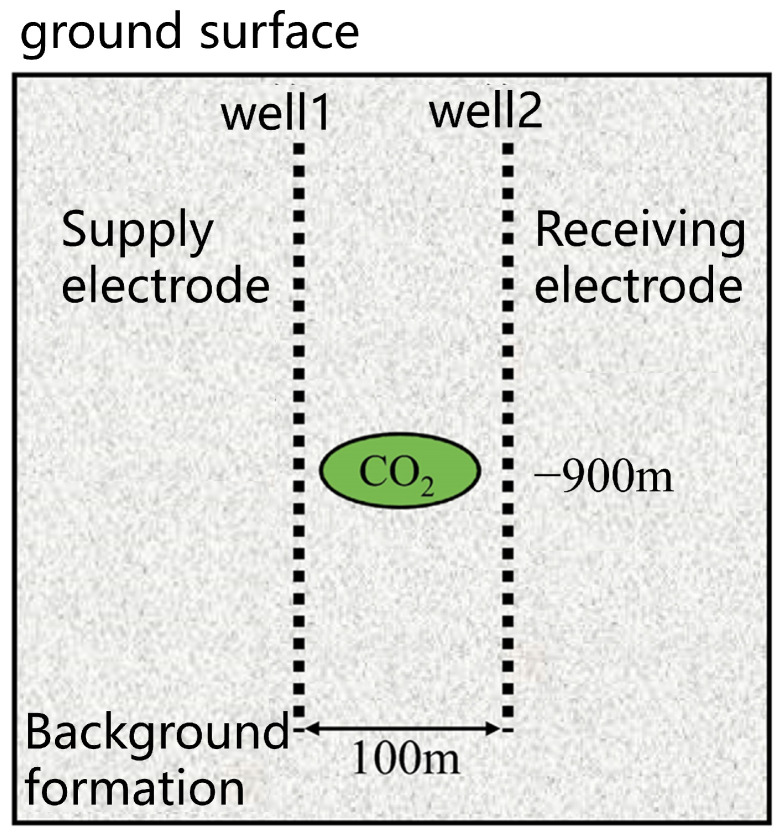
Geometry of the CO_2_ inversion model. The ellipsoidal storage region is centered between the two wells, simulating the CO_2_ injection zone at a depth of 900 m.

Therefore, this study establishes an inversion model for cross-borehole resistivity measurement (Figure 3). In the simulation process, the power supply electrode and receiving electrode are placed in observation wells 1 and 2, respectively. Typically, CO_2_ can reach a supercritical state at depths greater than 800 m; thus, we set the CO_2_ injection depth at 900 m. Notably, due to the cap rock’s barrier and the buoyancy of the gas, the injected CO_2_ typically forms a funnel-shaped distribution. To simplify this in the simulation, the CO_2_ injection zone is modeled as an ellipsoidal storage region with a constant aspect ratio, where variations in the ellipsoid sizes correspond to different volume ranges of the injected CO_2_. The electrodes are placed at depths between 800 m and 1000 m in two observation wells, located 50 m from the injection well, with the ellipsoidal storage region at the center. The power supply electrode in observation well 1 begins at a depth of 800 m and is moved downward in 10 m increments, while the potential values of the receiving electrode in observation well 2 are recorded. This results in a model with 21 × 21 apparent resistivity data points.

To investigate the changes in resistivity inversion imaging under different parameter conditions, two models were established: a homogeneous half-space model (model A) and a horizontally layered two-layer model (model B) [44,45]. Simulations were conducted by varying the CO_2_ storage zone volume, resistivity, and background resistivity. Additionally, simulations of the resistivity changes in the storage zone during CO_2_ diffusion were carried out for inversion imaging analysis. The imaging results were obtained by varying different variables of the storage zone, allowing an analysis of how these parameters influence the resistivity values. In the inversion results, the resistivity difference represents the deviation between the inverted resistivity values and the model’s set resistivity values. A comparative analysis was then carried out to evaluate the discrepancies between the inversion results and the model parameters. The model parameter settings are summarized in Table 1.

## 4. Study of the Inversion Effect of Storage Zone Resistivity

### 4.1. Influence of Storage Zone Volume Changes on Imaging Results

#### 4.1.1. Homogeneous Half-Space Model A1

**Figure 4:**
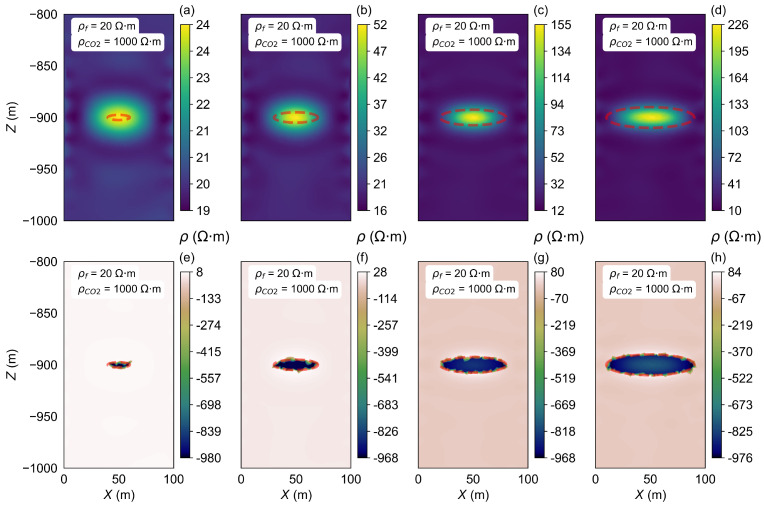
Inversion results of different $V_{{\mathrm{CO}}_{2}}$ in model A1. (**a**–**d**) show the resistivity inversion results at different stages of CO_2_ injection. Images (**e**–**h**) display the differences between the inverted resistivity values and the model parameters set for each stage. The red elliptical dashed line indicates the predefined storage zone.

As CO_2_ is continuously injected into the storage zone, the volume of the CO_2_ storage zone ($V_{{\mathrm{CO}}_{2}}$) increases. To assess the impact of these volume changes on the inverted resistivity results, the $V_{{\mathrm{CO}}_{2}}$ is varied at different stages of injection during the inversion process. The inversion results are presented in Figure 4.

The inverted background resistivity results generally align with the model settings, the red elliptical dashed line indicates the predefined storage zone, and the inverted location and dimensions of the storage zone are completely consistent with the model settings. The resistivity difference in the background layer region (Figure 4e–h) also confirms both the location and size of the storage zone. For example, as depicted in Figure 4a, the major axis, minor axis, and focal length of the CO_2_ storage zone are set at 10 m, 10 m, and 2.5 m, respectively. When the resistivity of the CO_2_ storage zone ($\rho_{{\mathrm{CO}}_{2}}$) is set to 1000 Ω·m, the inverted resistivity range falls between 19 and 24 Ω·m. The inverted resistivity near the storage zone is approximately 24 Ω·m, while the surrounding resistivity is inverted to 19 Ω·m, which is lower than the background resistivity ($\rho_{f}$) of 20 Ω·m. This is attributed to the high-resistance characteristics of the high-resistance storage zone, which results in reduced current density in the surrounding area. The significant discrepancy between the inverted resistivity of the storage zone and the model-set resistivity is primarily due to the relatively small $V_{{\mathrm{CO}}_{2}}$ and the $\rho_{f}$ contrast.

The difference in resistivity results obtained through inversion effectively reflects the resistivity variations. As the $V_{{\mathrm{CO}}_{2}}$ increases, the inverted resistivity of the storage zone gradually increases, with substantial variations, indicating that the $V_{{\mathrm{CO}}_{2}}$ has a large impact on the inversion outcomes. Although the resistivity value of the storage zone is set to be a relatively high value, small storage volumes make it challenging to obtain accurate inversion results.

#### 4.1.2. Horizontal Stratified Model B1

**Figure 5:**
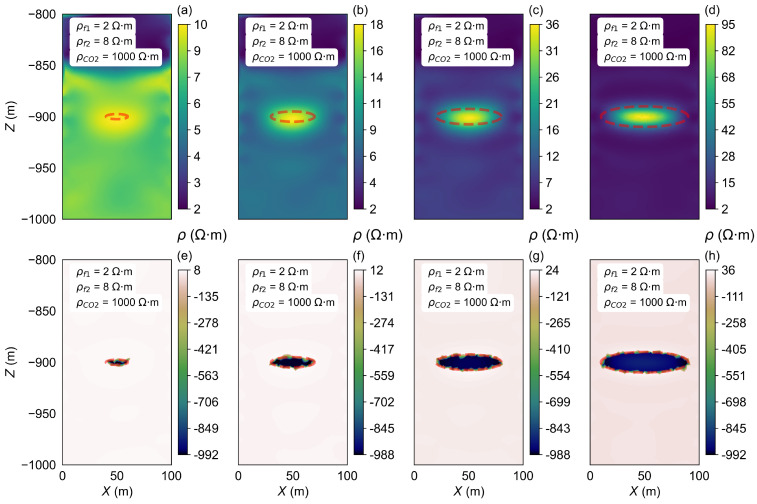
Inversion results of the $V_{{\mathrm{CO}}_{2}}$ changes in model B. (**a**–**d**) show the resistivity inversion results at different stages of CO_2_ injection. Images (**e**–**h**) display the differences between the inverted resistivity values and the model parameters set for each stage.

In most cases, the subsurface formations are typically horizontal and layered. To analyze the effect of varying $V_{{\mathrm{CO}}_{2}}$ on resistivity inversion under these conditions, a horizontal two-layer stratified model (model B1) is adopted. The model parameters used for this analysis are provided in Table 1. The inversion results for this model are shown in Figure 5.

For the horizontally layered model, when keeping the $\rho_{{\mathrm{CO}}_{2}}$ constant, as the CO_2_ injection volume increases, an increase in the CO_2_ injection volume leads to a gradual expansion of the storage zone. The inversion results, shown in Figure 5, clearly delineate the location and size of the storage zone, as indicated by the red dashed line. The inversion also reveals a clear horizontal layering effect, which is consistent with the accurate inversion of the background resistivity observed in model A1. As the $V_{{\mathrm{CO}}_{2}}$ increases, the inverted resistivity values of the storage zone gradually increase from 10 Ω·m to 95 Ω·m. This is approximately half of the value observed in model A1.

**Figure 6:**
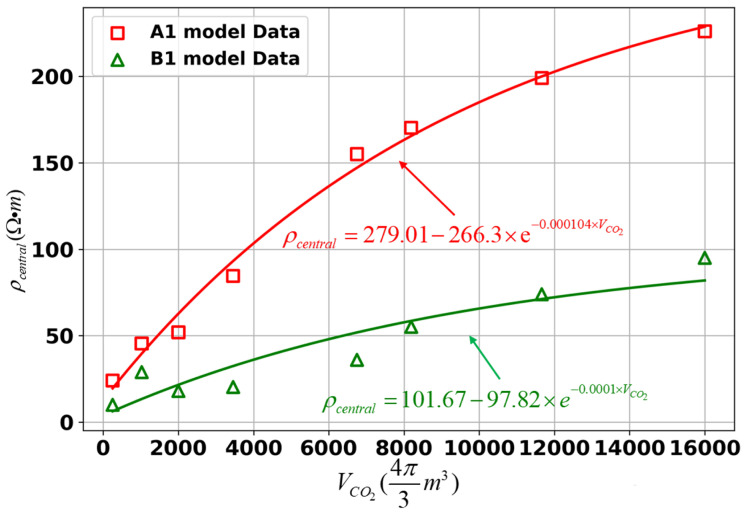
Relationship between the $V_{{\mathrm{CO}}_{2}}$ and $\rho_{central}$ in point (50, −900) for different models. The corresponding lines represent the best-fit exponential functions.

For both model A1 and model B1, we analyze the relationship between the $V_{{\mathrm{CO}}_{2}}$ and the inverted resistivity of the central storage zone ($\rho_{central}$), i.e., the point of (50, −900) (Figure 6). The results indicate that, in both models, the $\rho_{central}$ increases as the $V_{{\mathrm{CO}}_{2}}$ increases. The fitting curves approximate exponential functions, with the growth rate of $\rho_{central}$ gradually decreasing as the $V_{{\mathrm{CO}}_{2}}$ increases. However, the rate of decrease slows down, and the $\rho_{central}$ ultimately approaches a stable value (e.g., approximately 279.01 Ω·m in model A1). Further research on this will be conducted in subsequent sections.

### 4.2. Influence of Storage Zone Resistivity Changes on Imaging Results

#### 4.2.1. Homogeneous Half-Space Model A2

**Figure 7:**
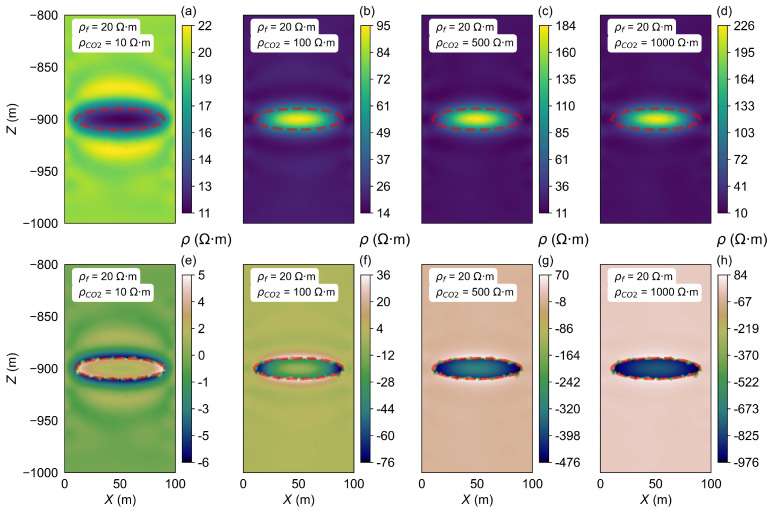
Inversion results of the $\rho_{{\mathrm{CO}}_{2}}$ changes in model A2. (**a**–**d**) show the resistivity inversion results at different stages of CO_2_ injection. Images (**e**–**h**) display the differences between the inverted resistivity values and the model parameters set for each stage.

As the injection time increases, once the $V_{{\mathrm{CO}}_{2}}$ reaches the set maximum capacity, continued CO_2_ injection will cause significant changes in the resistivity within the storage zone. The inversion process was carried out for the homogeneous half-space model A2 to investigate this effect. The background resistivity was set to 20 Ω·m, while the semi-major axis, semi-minor axis, and focal length of the ellipsoidal CO_2_ storage zone were defined as 40 m, 40 m, and 10 m, respectively. Four sets of $\rho_{{\mathrm{CO}}_{2}}$ were considered, 10 Ω·m, 100 Ω·m, 500 Ω·m, and 1000 Ω·m, as outlined in the parameters presented in Table 1. The inversion results are shown in Figure 7.

As CO_2_ continues to be injected into the storage zone, when keeping the $V_{{\mathrm{CO}}_{2}}$ constant, the $\rho_{{\mathrm{CO}}_{2}}$ gradually changes. This results in varying resistivity inversion images, as shown in Figure 7.

In Figure 7a, the resistivity of the storage zone is set to 10 Ω·m. The inverted resistivity of the CO_2_ storage zone inversion result close to 11 Ω·m and the surrounding areas have resistivity values close to 20 Ω·m, which matches the model’s parameters. However, a notable change occurs when the $\rho_{{\mathrm{CO}}_{2}}$ exceeds the $\rho_{f}$. In this scenario, the resistivity inversion results become less accurate as the contrast between the storage zone and surrounding layers increases (Figure 7b–d). In Figure 7d, the $\rho_{{\mathrm{CO}}_{2}}$ is set to 1000 Ω·m, while the inverted resistivity of the storage zone resistivity is only 226 Ω·m. In all cases of the existence of the high-resistivity anomaly, the inverted resistivity results show a low-resistivity surrounding layer around the high-resistivity storage zone. The differences between the inversion results and the model settings are shown in Figure 7e–h, which can clearly reflect the individual impact of the storage zone on the inversion results. As the resistivity increases (from 10 Ω·m to 500 Ω·m), the difference becomes more pronounced.

In general, our results show that when the storage zone resistivity is much greater than the background resistivity, the inversion process may underestimate the resistivity in the storage zone, as the system’s electrical response becomes more sensitive to the larger resistivity contrast. This discrepancy leads to less precise inverted results, as demonstrated in the cases where the resistivity of the storage zone exceeds the background value significantly.

#### 4.2.2. Horizontal Two-Layer Model B2

**Figure 8:**
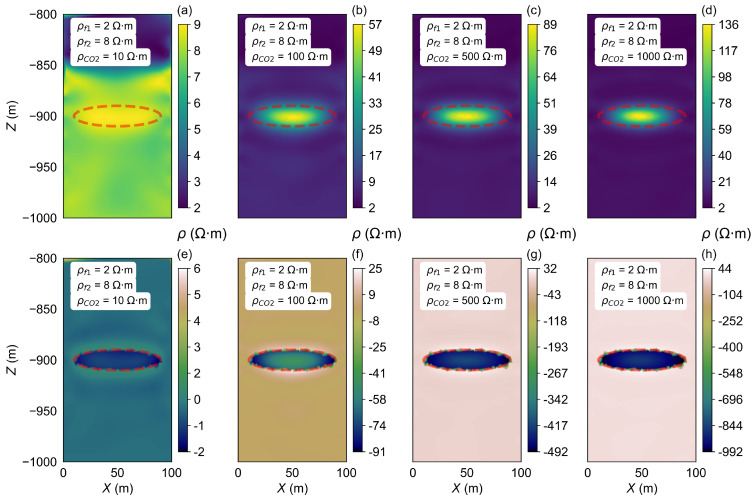
Inversion results of the $\rho_{{\mathrm{CO}}_{2}}$ changes in model B2. (**a**–**d**) show the resistivity inversion results at different stages of CO_2_ injection. Images (**e**–**h**) display the differences between the inverted resistivity values and the model parameters set for each stage.

The horizontal two-layer model B2 was established, and the $\rho_{{\mathrm{CO}}_{2}}$ was changed, with values from 10 Ω·m to 1000 Ω·m. The inversion results are shown in Figure 8.

In contrast to the findings in model A1, where the storage zone resistivity can be accurately inverted to match the model parameters within a certain range, the results from model B2 indicate a different trend. Specifically, only when the $\rho_{{\mathrm{CO}}_{2}}$ is set to 10 Ω·m do the inversion results match the model’s set value of 10 Ω·m. As the $\rho_{{\mathrm{CO}}_{2}}$ increases, the inverted resistivity values gradually rise; however, even at the highest $\rho_{{\mathrm{CO}}_{2}}$ value of 1000 Ω·m, the inverted resistivity only reaches a maximum of 136 Ω·m, which is still significantly lower than the model’s set value of 1000 Ω·m.

**Figure 9:**
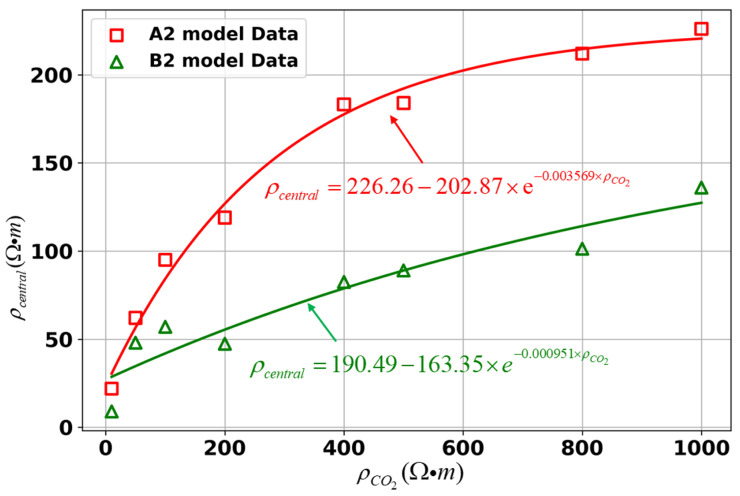
Relationship between the $\rho_{{\mathrm{CO}}_{2}}$ and the $\rho_{central}$ for different models.

To further investigate the relationship between the $\rho_{{\mathrm{CO}}_{2}}$ and the $\rho_{central}$ in both models, fitting curves for different models were plotted as shown in Figure 9. The results indicate that, overall, as the $\rho_{{\mathrm{CO}}_{2}}$ increases, the $\rho_{central}$ also gradually increases. The fitting curves for both model A2 and model B2 approximate exponential functions. When only the $\rho_{{\mathrm{CO}}_{2}}$ is varied, a notable deviation between the central resistivity and the set values occurs, with this discrepancy becoming more pronounced as the $\rho_{{\mathrm{CO}}_{2}}$ increases.

### 4.3. Impact of Background Resistivity Variations on Imaging Results

#### 4.3.1. Homogeneous Half-Space Model A3

**Figure 10:**
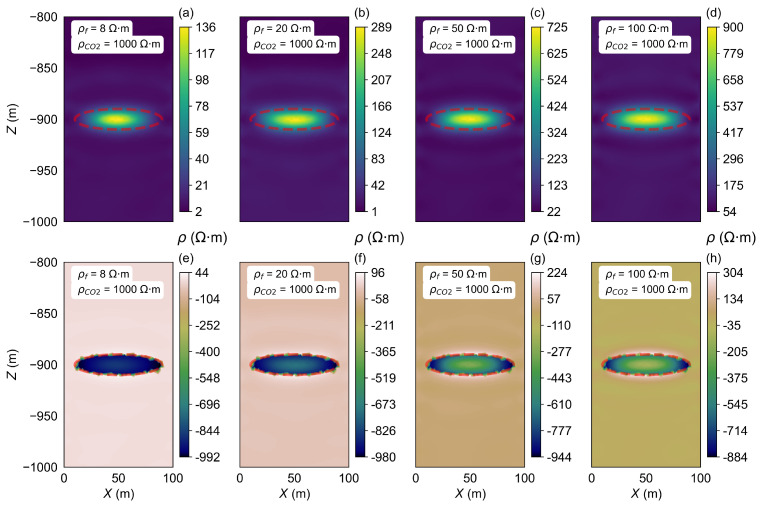
Inversion results of the $\rho_{f}$ changes in model A3. (**a**–**d**) show the resistivity inversion results at different stages of CO_2_ injection. Images (**e**–**h**) display the differences between the inverted resistivity values and the model parameters set for each stage.

As mentioned above, we have discussed the effects of $V_{{\mathrm{CO}}_{2}}$ and $\rho_{{\mathrm{CO}}_{2}}$ variations on the inversion results. It was found that, compared to the uniform half-space model, the resistivity values from the inversion are significantly different in the horizontally layered model. This discrepancy could be due to the influence of the $\rho_{f}$ settings. Therefore, in this section, we will analyze the changes in inversion resistivity by altering the $\rho_{f}$ in model A3. The model parameters are set as shown in Table 1, and the inversion results are shown in Figure 10.

In all four cases, the $\rho_{f}$ is set differently, but the resistivity difference in the background region is close to 0, indicating that the inverted background resistivity matches the model parameters. The position and size of the storage zone obtained from the inversion are in perfect agreement with the model settings. Since the $\rho_{f}$ is relatively low, the inverted resistivity of the high-resistance storage zone is also lower. Figure 10e–h show that as the $\rho_{f}$ increases, the resistivity difference also increases, reflecting similar trends to the inverted resistivity values.

The discrepancy between the inverted resistivity and the model settings in the high-resistance storage zone is mainly attributed to the influence of the $\rho_{f}$. When the $\rho_{f}$ is increased to 100 Ω·m, the change in the inverted storage zone resistivity is relatively small. This suggests that once the $\rho_{f}$ reaches a certain threshold, its impact tends to stabilize.

#### 4.3.2. Horizontal Two-Layered Model B3

Typically, the cap layer of a CO_2_ storage zone consists of low-resistivity rock structures, which are better for geological storage. Therefore, in this study of the horizontal model B3, the resistivity of the first layer (cap layer) remains constant, and only the resistivity of the second layer (storage layer) is varied.

**Figure 11:**
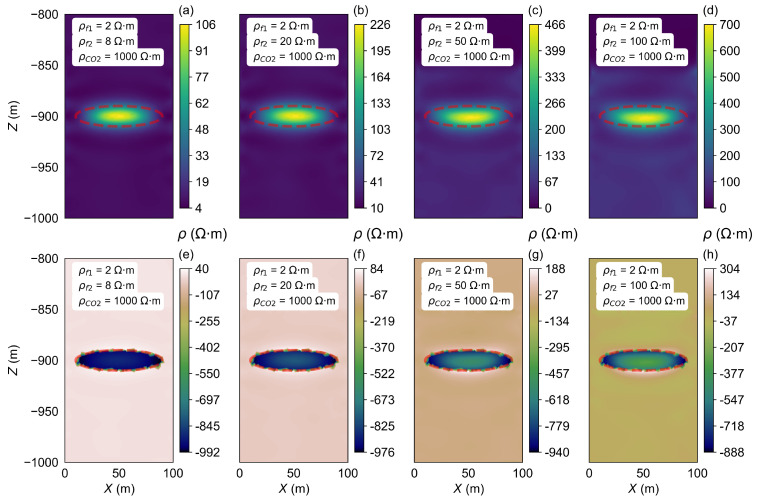
Inversion results of the $\rho_{f}$ changes in model B3. (**a**–**d**) show the resistivity inversion results at different stages of CO_2_ injection. Images (**e**–**h**) display the differences between the inverted resistivity values and the model parameters set for each stage.

Similarly, the location of the storage zone is accurately reflected. Moreover, the background resistivity is correctly inverted in all four models. As the $\rho_{f}$ increases, the inverted resistivity of the storage zone continues to show a consistently high growth trend. Compared to model A3, the inverted resistivity values for model B3 are lower. This is because the presence of the first low-resistivity layer causes the current density in the storage zone to be relatively sparse, leading to lower potential in the forward modeling process (see Figure 11).

**Figure 12:**
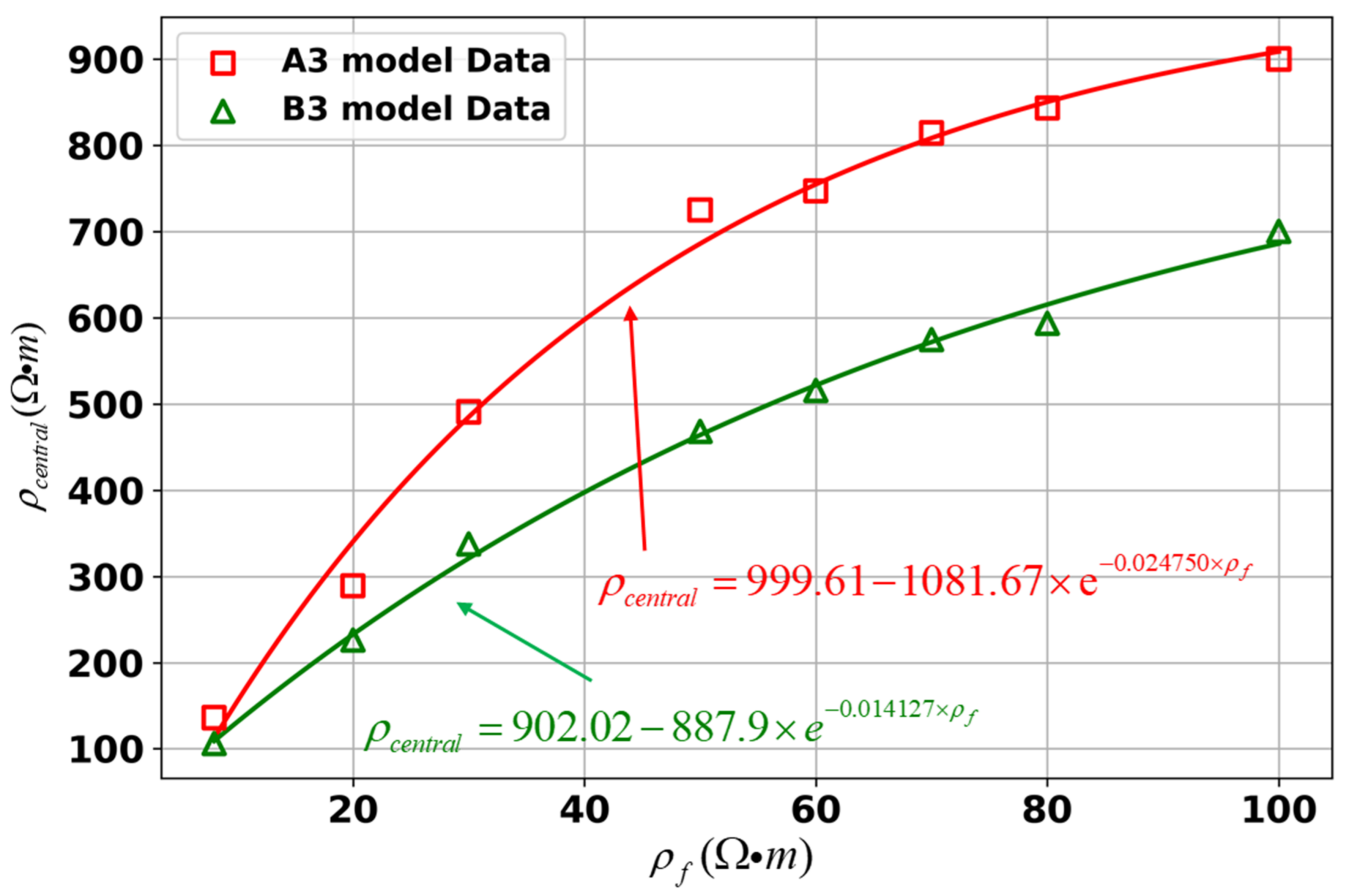
Relationship between the $\rho_{f}$ and the $\rho_{central}$ for different models.

By analyzing the $\rho_{central}$ variations in both models under different $\rho_{f}$ conditions, as shown in Figure 12, it is further demonstrated that changes in the $\rho_{f}$ influence the inversion results. Under model A3 conditions, the inverted resistivity ultimately converges to 999.61 Ω·m, which is very close to the set CO_2_ resistivity of 1000 Ω·m. Similarly, model B3 reaches a final resistivity of 902.02 Ω·m. These results indicate that the $\rho_{f}$ is a key factor affecting the inversion resistivity distribution.

From the analysis of the three factors—storage zone volume, resistivity, and background resistivity—it is found that the impact of background resistivity on the inversion result is greater than the impact of storage zone resistivity, which, in turn, is greater than the impact of storage zone volume. This finding underscores the importance of conducting a thorough pre-analysis of the background strata prior to monitoring the CO_2_ injection and storage process in practical CO_2_ sequestration operations.

### 4.4. Study on the Impact of CO_2_ Diffusion on Imaging Results

As the CO_2_ injection time increases, the CO_2_ content in the storage zone gradually increases, and the volume occupied by CO_2_ expands. Once CO_2_ is completely distributed within the storage zone, the resistivity will gradually decrease. The changes in resistivity during this process, as discussed earlier, are due to the injection of CO_2_ as a high-pressure gas, which alters the elastic properties of the reservoir. CO_2_ will diffuse to some extent, and this diffusion will affect the properties of the subsurface region as well as surface water, potentially posing risks to both the ecosystem and human activities. Therefore, it is essential to closely analyze the CO_2_ diffusion process and its implications for the monitoring and safety of CO_2_ sequestration operations.

In the previous model, the ratio of the semi-axes of the elliptical storage zone was 4:4:1, with a relatively small width in the vertical direction (z-axis). To improve the inversion performance in the vertical direction, the semi-axis ratio of the elliptical shape is set to 2:2:1 for the CO_2_ diffusion simulation. This change allows for a more representative model of the CO_2_ diffusion within the subsurface, ensuring better spatial resolution in the vertical dimension, which is critical for assessing the behavior of CO_2_ as it migrates within the geological formations.

**Figure 13:**
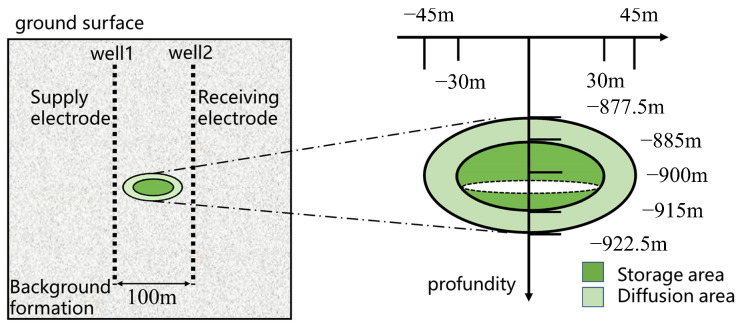
CO_2_ diffusion model geometry. The smaller ellipse represents the storage zone and the larger ellipse indicates the diffusion zone.

The simulation assumes that no diffusion occurs during the initial injection stage, where the CO_2_ volume is still low. Diffusion is set to begin only after the storage zone is fully filled with CO_2_. In this model, the semi-axes of the small elliptical storage zone are set to 30 m, 30 m, and 15 m, and these values are kept constant throughout the simulation. These dimensions represent the storage zone before diffusion begins, and they reflect the typical spatial extent of CO_2_ accumulation in the initial stages of injection. Once diffusion begins, the diffusion area is defined by a larger elliptical shape, with semi-axes set to 45 m, 45 m, and 22.5 m, corresponding to the maximum extent of CO_2_ distribution. The region between the small and large ellipses is designated as the diffusion zone, as illustrated in Figure 13. This area represents the region where CO_2_ gradually spreads from the initial storage zone into the surrounding rock formations. The rate of resistivity change within the diffusion zone depends on factors such as the rate of CO_2_ injection, the permeability of the rock, and the diffusion characteristics of CO_2_ in the subsurface. To simplify, the diffusion process is modeled by gradually reducing the resistivity in this zone, as CO_2_ replaces the air or water that was originally present in the pore spaces of the geological formations. The resistivity of the diffusion area is set as follows according to Figure 13:

(13) $$\rho_{escape}=0.9\times \rho_{f}+0.1\times \rho_{{\mathrm{CO}}_{2}}$$

During the CO_2_ diffusion process, with the storage volume held constant, the resistivity inversion results are analyzed for different injection periods (i.e., varying resistivity of the CO_2_ storage and diffusion zone). First, the homogeneous half-space model A4 is established, with the $\rho_{{\mathrm{CO}}_{2}}$ set at 10, 100, 500, and 1000 Ω·m, and the resistivity of the diffusion zone set at 19, 28, 68, and 118 Ω·m.

**Figure 14:**
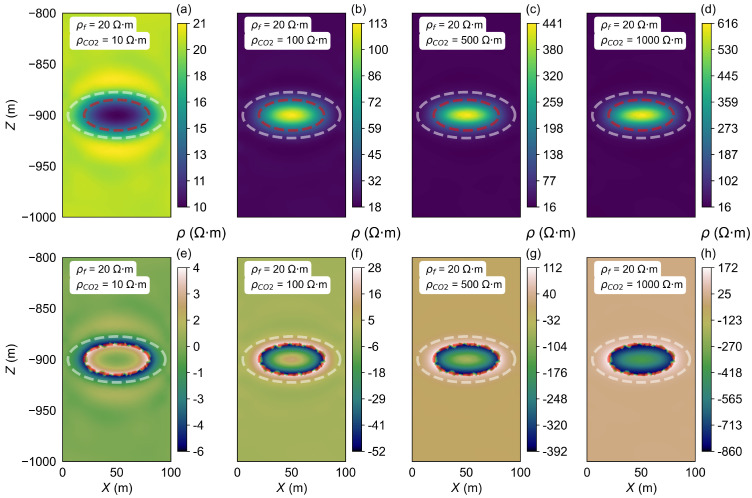
Inversion results of changing the storage area when CO_2_ diffuses in model A4. (**a**–**d**) show the resistivity inversion results at different stages of CO_2_ injection. Images (**e**–**h**) display the differences between the inverted resistivity values and the model parameters set for each stage. The red elliptical dashed line indicates the location of the storage zone, and the area between the red dashed line and the white elliptical dashed line represents the diffusion zone.

Figure 14 shows the inversion results after diffusion. As the $\rho_{{\mathrm{CO}}_{2}}$ increases, the inversion results show corresponding changes. However, it is challenging to directly distinguish the differences in the diffusion zone’s range from the inversion results alone. To further investigate the impact of diffusion on the inversion results, the differences between the inverted resistivity after diffusion and the initial model before diffusion (which can be measured before CO_2_ injection) are calculated (Figure 14e–h). By comparing the inversion results to the initial model settings, we can observe that significant layering appears in the resistivity values of both the sequestration and diffusion zones, as indicated by the areas marked by white dashed and red dashed lines. For example, Figure 14h shows a significant increase in the resistivity difference before and after diffusion. This is because the diffusion zone resistivity of 118 Ω·m is much higher than the model’s initial settings. The resistivity difference in the diffusion zone is close to 118 Ω·m, enabling an accurate identification of the diffusion region and its corresponding resistivity values.

The difference results highlight the effects of CO_2_ diffusion on the resistivity distribution, allowing for a more accurate identification of the diffusion region and providing deeper insights into the impact of CO_2_ on subsurface resistivity. This analysis emphasizes the importance of considering diffusion effects when monitoring CO_2_ sequestration and tracking plume migration in real-world applications.

**Figure 15:**
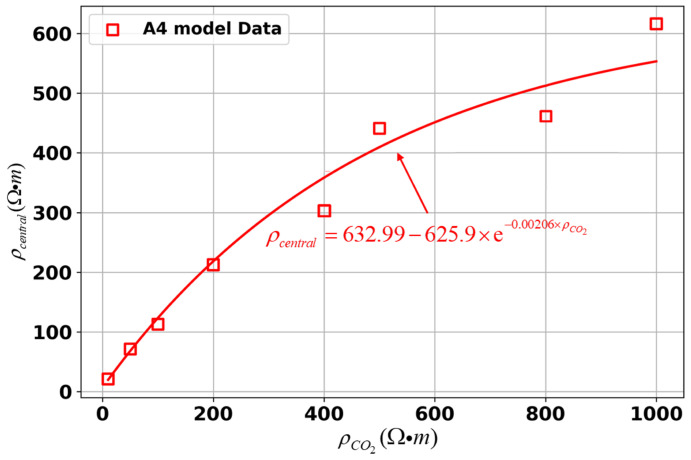
Relationship between the $\rho_{{\mathrm{CO}}_{2}}$ and the $\rho_{central}$ for different models under diffusion conditions.

We also analyze the variation in the $\rho_{central}$ under diffusion conditions for both models, as shown in Figure 15. The background resistivity plays a key role in influencing the diffusion behavior and, consequently, the resistivity inversion results. However, overall, under diffusion conditions, the $\rho_{central}$ increases as the resistivity of the sequestration zone increases, verifying the accuracy of the inversion. The increase in $\rho_{central}$ under diffusion conditions is consistent with the expectation that CO_2_ injection reduces the $\rho_{{\mathrm{CO}}_{2}}$ due to the displacement of pore fluids by CO_2_. As the diffusion process progresses, the injected CO_2_ gradually fills the storage zone, leading to a redistribution of resistivity.

## 5. Conclusions

This study employs the IGN method to examine the resistivity inversion of CO_2_ sequestration zones using cross-borehole measurements. We constructed two geological models—a homogeneous half-space model and a horizontally layered model—to evaluate the impacts of storage zone volume, storage zone resistivity, background resistivity, and CO_2_ diffusion on inversion results. Increasing storage zone volume directly correlates with a rise in inverted resistivity; however, as volume grows, the rate of increase slows and stabilizes at a specific value, reflecting a size-dependent limit. The resistivity of the storage zone significantly influences inversion accuracy; reliable outcomes are achieved within a defined range (e.g., below 100 Ω·m in the homogeneous model), but when this threshold is exceeded, substantial deviations occur, with errors escalating as resistivity rises further. Background resistivity variations notably affect inversion results, particularly in high-resistivity zones, though this effect plateaus beyond a saturation point slightly above 100 Ω·m, beyond which additional increases have minimal impact. When CO_2_ diffusion is introduced, a distinct transition zone emerges between the storage and background layers. Following CO_2_ injection, resistivity decreases in both the storage and diffusion zones, driving pronounced changes in the inverted resistivity. Comparisons of pre- and post-injection data enable clear identification of the diffusion zone, enhancing tracking capabilities. This study thus provides a reliable approach to ensuring the safety and stability of CO_2_ storage operations, with practical implications for optimizing long-term sequestration strategies.

## Figures and Tables

**Table 1:** Inversion model setup parameters.

Influence Factor	Different Models	Figure Number	Layer Resistivity (Ω·m)	CO_2_ Volume ($\times \frac{4\pi }{3}$ m^3^)	CO_2_ Resistivity (Ω·m)	Imaging Results
Storage Zone Volume	A1	(a)∼(d)	20 (0∼2000 m)	250	1000	Resistivity
		(e)∼(h)		2000		Resistivity Difference
Storage Zone Volume	B1	(a)∼(d)	First Layer: 2 (0∼850 m)	6750		Resistivity
		(e)∼(h)	Second Layer: 8 (850∼2000 m)	16,000		Resistivity Difference
Storage Zone Resistivity	A2	(a)∼(d)	2020 (0∼2000 m)	16,000	10	Resistivity
		(e)∼(h)			100	Resistivity Difference
Storage Zone Resistivity	B2	(a)∼(d)	First Layer: 2 (0∼850 m)		500	Resistivity
		(e)∼(h)	Second Layer: 8 (850∼2000 m)		1000	Resistivity Difference
Background Resistivity	A3	(a)∼(d)	8, 20, 50, 100	16,000	1000	Resistivity
		(e)∼(h)				Resistivity Difference
Background Resistivity	B3	(a)∼(d)	First Layer: 2 (0∼850 m)			Resistivity
		(e)∼(h)	Second Layer: 8 (850∼2000 m)			Resistivity Difference
CO_2_ Diffusion in Storage Zone Resistivity	A4	(a)∼(d)	20	Storage Zone/ Diffusion Zone (13,500/ 32,063)	Storage Zone 10/19 100/28 500/68 1000/118	Resistivity
		(e)∼(h)				Resistivity Difference

## Data Availability

Data are contained within the article.
